# 6-Chloro­quinolin-2(1*H*)-one

**DOI:** 10.1107/S1600536811053359

**Published:** 2011-12-21

**Authors:** Chen-Guang Zhang, Yang-Hui Luo

**Affiliations:** a Ordered Matter Science Research Center, College of Chemistry and Chemical Engineering, Southeast University, Nanjing 210096, People’s Republic of China

## Abstract

In the title compound, C_9_H_6_ClNO, the Cl atom deviates by 0.142 (1) Å from the quinoline ring mean plane (r.m.s. deviation = 0.013 Å). In the crystal, N—H⋯O hydrogen bonds link the mol­ecules into [010] *C*(4) chains. Aromatic π–π stacking inter­actions [shortest centroid⋯centroid distance = 3.685 (3) Å] are also observed.

## Related literature

For background to quinoline derivatives as pharmaceuticals, see: Luo *et al.* (2011 ▶).
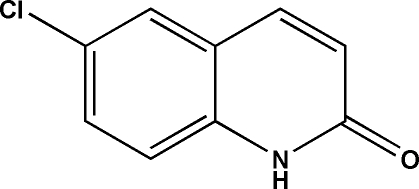


## Experimental

### 

#### Crystal data

C_9_H_6_ClNO
*M* *_r_* = 179.60
Orthorhombic, 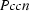

*a* = 24.951 (19) Å
*b* = 7.733 (6) Å
*c* = 7.988 (6) Å
*V* = 1541 (2) Å^3^
*Z* = 8
Mo *K*α radiation
μ = 0.44 mm^−1^
*T* = 296 K
0.20 × 0.20 × 0.20 mm

#### Data collection

Rigaku SCXmini CCD diffractometer
Absorption correction: multi-scan (*CrystalClear*; Rigaku, 2005 ▶) *T*_min_ = 0.917, *T*_max_ = 0.917
9911 measured reflections
1353 independent reflections
1161 reflections with *I* > 2σ(*I*)
*R*_int_ = 0.024

#### Refinement

*R*[*F* ^2^ > 2σ(*F* ^2^)] = 0.030
*wR*(*F* ^2^) = 0.088
*S* = 1.06
1353 reflections
113 parameters
H atoms treated by a mixture of independent and constrained refinement
Δρ_max_ = 0.17 e Å^−3^
Δρ_min_ = −0.23 e Å^−3^

### 

Data collection: *CrystalClear* (Rigaku, 2005 ▶); cell refinement: *CrystalClear*; data reduction: *CrystalClear*; program(s) used to solve structure: *SHELXS97* (Sheldrick, 2008 ▶); program(s) used to refine structure: *SHELXL97* (Sheldrick, 2008 ▶); molecular graphics: *DIAMOND* (Brandenburg & Putz, 2005 ▶); software used to prepare material for publication: *SHELXL97*.

## Supplementary Material

Crystal structure: contains datablock(s) I, global. DOI: 10.1107/S1600536811053359/hb6549sup1.cif

Structure factors: contains datablock(s) I. DOI: 10.1107/S1600536811053359/hb6549Isup2.hkl

Supplementary material file. DOI: 10.1107/S1600536811053359/hb6549Isup3.cml

Additional supplementary materials:  crystallographic information; 3D view; checkCIF report

## Figures and Tables

**Table 1:** Hydrogen-bond geometry (Å, °)

*D*—H⋯*A*	*D*—H	H⋯*A*	*D*⋯*A*	*D*—H⋯*A*
N1—H1⋯O1^i^	0.887 (19)	1.98 (2)	2.859 (2)	168.7 (17)

Symmetry code: (i) 
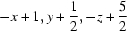
.

**Fig. 1.**
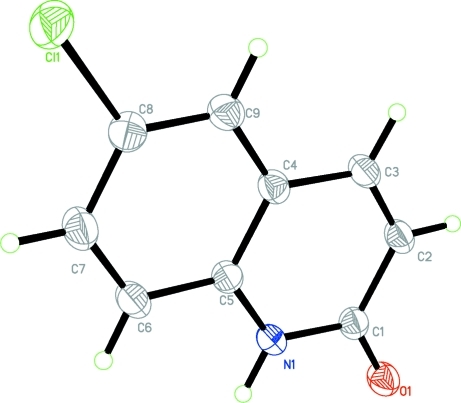
The molecular structure of the title compound with displacement ellipsoids drawn at the 30% probability level.

**Fig. 2.**
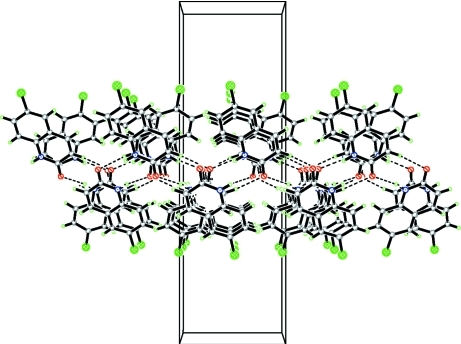
A packing view down the a axis showing hydrogen bonds as dashed lines.

## supplementary crystallographic information

### Experimental

The title compound was purchased from ChemFuture PharmaTech, Ltd (Nanjing, Jiangsu). Pink prisms were obtained by slow evaporation of a methanol solution.

### Refinement

All H atoms attached to C atoms and O atoms were fixed geometrically and treated as riding with C—H = 0.93 Å (CH) and N—H = 0.86 Å with *U*_iso_(H) = 1.2*U*_eq_.

### Crystal data

**Table:**

C_9_H_6_ClNO	*F*(000) = 736
*M**_r_* = 179.60	*D*_x_ = 1.548 Mg m^−^^3^
Orthorhombic, *P**c**c**n*	Mo *K*α radiation, λ = 0.71073 Å
Hall symbol: -P 2ab 2ac	Cell parameters from 1357 reflections
*a* = 24.951 (19) Å	θ = 1.6–25.0°
*b* = 7.733 (6) Å	µ = 0.44 mm^−^^1^
*c* = 7.988 (6) Å	*T* = 296 K
*V* = 1541 (2) Å^3^	Prism, pink
*Z* = 8	0.20 × 0.20 × 0.20 mm

### Data collection

**Table:**

Rigaku SCXmini CCD diffractometer	1353 independent reflections
Radiation source: fine-focus sealed tube	1161 reflections with *I* > 2σ(*I*)
graphite	*R*_int_ = 0.024
Detector resolution: 13.6612 pixels mm^-1^	θ_max_ = 25.0°, θ_min_ = 1.6°
CCD_Profile_fitting scans	*h* = −29→29
Absorption correction: multi-scan (*CrystalClear*; Rigaku, 2005)	*k* = −9→9
*T*_min_ = 0.917, *T*_max_ = 0.917	*l* = −8→9
9911 measured reflections	

### Refinement

**Table:**

Refinement on *F*^2^	Primary atom site location: structure-invariant direct methods
Least-squares matrix: full	Secondary atom site location: difference Fourier map
*R*[*F*^2^ > 2σ(*F*^2^)] = 0.030	Hydrogen site location: inferred from neighbouring sites
*wR*(*F*^2^) = 0.088	H atoms treated by a mixture of independent and constrained refinement
*S* = 1.06	*w* = 1/[σ^2^(*F*_o_^2^) + (0.0469*P*)^2^ + 0.4479*P*] where *P* = (*F*_o_^2^ + 2*F*_c_^2^)/3
1353 reflections	(Δ/σ)_max_ = 0.001
113 parameters	Δρ_max_ = 0.17 e Å^−^^3^
0 restraints	Δρ_min_ = −0.23 e Å^−^^3^

### Special details

**Table:**

Geometry. All e.s.d.'s (except the e.s.d. in the dihedral angle between two l.s. planes) are estimated using the full covariance matrix. The cell e.s.d.'s are taken into account individually in the estimation of e.s.d.'s in distances, angles and torsion angles; correlations between e.s.d.'s in cell parameters are only used when they are defined by crystal symmetry. An approximate (isotropic) treatment of cell e.s.d.'s is used for estimating e.s.d.'s involving l.s. planes.
Refinement. Refinement of *F*^2^ against ALL reflections. The weighted *R*-factor *wR* and goodness of fit *S* are based on *F*^2^, conventional *R*-factors *R* are based on *F*, with *F* set to zero for negative *F*^2^. The threshold expression of *F*^2^ > σ(*F*^2^) is used only for calculating *R*-factors(gt) *etc*. and is not relevant to the choice of reflections for refinement. *R*-factors based on *F*^2^ are statistically about twice as large as those based on *F*, and *R*- factors based on ALL data will be even larger.

### Fractional atomic coordinates and isotropic or equivalent isotropic displacement parameters (Å^2^)

**Table:**

	*x*	*y*	*z*	*U*_iso_*/*U*_eq_	
Cl1	0.73621 (2)	1.01875 (7)	0.69204 (7)	0.0614 (2)	
N1	0.54956 (5)	0.88864 (17)	1.12378 (17)	0.0369 (3)	
O1	0.49027 (5)	0.70991 (15)	1.25021 (16)	0.0472 (3)	
C1	0.53024 (6)	0.7277 (2)	1.1598 (2)	0.0369 (4)	
C5	0.59440 (6)	0.9205 (2)	1.02710 (19)	0.0349 (4)	
C2	0.55943 (7)	0.5842 (2)	1.0882 (2)	0.0398 (4)	
H2	0.5477	0.4719	1.1079	0.048*	
C4	0.62280 (6)	0.7806 (2)	0.95924 (19)	0.0357 (4)	
C9	0.66766 (7)	0.8129 (2)	0.8591 (2)	0.0409 (4)	
H9	0.6871	0.7214	0.8140	0.049*	
C7	0.65567 (7)	1.1184 (2)	0.8970 (2)	0.0487 (5)	
H7	0.6671	1.2308	0.8759	0.058*	
C8	0.68283 (7)	0.9794 (2)	0.8280 (2)	0.0426 (4)	
C3	0.60308 (7)	0.6101 (2)	0.9940 (2)	0.0404 (4)	
H3	0.6211	0.5151	0.9500	0.049*	
C6	0.61155 (7)	1.0894 (2)	0.9970 (2)	0.0453 (4)	
H6	0.5933	1.1821	1.0443	0.054*	
H1	0.5337 (8)	0.980 (2)	1.170 (2)	0.044 (5)*	

### Atomic displacement parameters (Å^2^)

**Table:**

	*U*^11^	*U*^22^	*U*^33^	*U*^12^	*U*^13^	*U*^23^
Cl1	0.0512 (3)	0.0595 (3)	0.0736 (4)	0.0024 (2)	0.0190 (2)	0.0143 (2)
N1	0.0428 (8)	0.0276 (7)	0.0404 (8)	0.0031 (6)	0.0035 (6)	−0.0012 (6)
O1	0.0483 (7)	0.0359 (7)	0.0574 (8)	−0.0015 (5)	0.0130 (7)	0.0020 (6)
C1	0.0406 (9)	0.0330 (9)	0.0371 (9)	−0.0011 (7)	−0.0033 (7)	0.0012 (7)
C5	0.0383 (8)	0.0322 (8)	0.0342 (8)	0.0014 (7)	−0.0020 (7)	−0.0001 (7)
C2	0.0470 (10)	0.0269 (8)	0.0454 (9)	0.0006 (7)	−0.0023 (8)	−0.0004 (7)
C4	0.0394 (8)	0.0317 (9)	0.0359 (9)	0.0045 (6)	−0.0050 (7)	0.0004 (7)
C9	0.0399 (9)	0.0400 (9)	0.0428 (10)	0.0078 (7)	−0.0007 (8)	−0.0004 (8)
C7	0.0499 (10)	0.0343 (9)	0.0618 (11)	−0.0035 (8)	0.0056 (9)	0.0036 (9)
C8	0.0368 (9)	0.0450 (10)	0.0461 (10)	0.0016 (7)	0.0009 (7)	0.0049 (8)
C3	0.0475 (10)	0.0298 (8)	0.0440 (10)	0.0078 (7)	−0.0022 (8)	−0.0025 (7)
C6	0.0506 (10)	0.0294 (9)	0.0558 (11)	0.0031 (7)	0.0071 (9)	−0.0024 (8)

### Geometric parameters (Å, °)

**Table:**

Cl1—C8	1.745 (2)	C4—C9	1.398 (2)
N1—C1	1.365 (2)	C4—C3	1.434 (2)
N1—C5	1.382 (2)	C9—C8	1.365 (3)
N1—H1	0.887 (19)	C9—H9	0.9300
O1—C1	1.239 (2)	C7—C6	1.379 (3)
C1—C2	1.445 (2)	C7—C8	1.385 (3)
C5—C6	1.395 (2)	C7—H7	0.9300
C5—C4	1.402 (2)	C3—H3	0.9300
C2—C3	1.339 (2)	C6—H6	0.9300
C2—H2	0.9300		
			
C1—N1—C5	124.49 (14)	C8—C9—C4	119.66 (15)
C1—N1—H1	118.7 (12)	C8—C9—H9	120.2
C5—N1—H1	116.7 (12)	C4—C9—H9	120.2
O1—C1—N1	120.54 (15)	C6—C7—C8	119.65 (16)
O1—C1—C2	123.46 (15)	C6—C7—H7	120.2
N1—C1—C2	116.00 (15)	C8—C7—H7	120.2
N1—C5—C6	120.77 (14)	C9—C8—C7	121.59 (17)
N1—C5—C4	119.20 (14)	C9—C8—Cl1	119.31 (14)
C6—C5—C4	120.03 (16)	C7—C8—Cl1	119.06 (14)
C3—C2—C1	121.17 (15)	C2—C3—C4	121.70 (15)
C3—C2—H2	119.4	C2—C3—H3	119.2
C1—C2—H2	119.4	C4—C3—H3	119.2
C9—C4—C5	119.21 (15)	C7—C6—C5	119.83 (16)
C9—C4—C3	123.33 (15)	C7—C6—H6	120.1
C5—C4—C3	117.44 (15)	C5—C6—H6	120.1

### Hydrogen-bond geometry (Å, °)

**Table:**

*D*—H···*A*	*D*—H	H···*A*	*D*···*A*	*D*—H···*A*
N1—H1···O1^i^	0.887 (19)	1.98 (2)	2.859 (2)	168.7 (17)

Symmetry codes: (i) −*x*+1, *y*+1/2, −*z*+5/2.
